# The effect of acceptance and commitment therapy on communication avoidance, emotional well-being, and hyperarousal in Iranian caregivers of women with advanced breast cancer

**DOI:** 10.1007/s44192-025-00238-x

**Published:** 2026-06-25

**Authors:** Manijeh Firoozi, Mehdi Sharifi, Christopher Alan Lewis, Atefeh Arabi, Umm al-Banīn Esmati, Masoumeh Tadayyoni, David Lester, Hanifa Saeed Aboubakr

**Affiliations:** 1 Department of Psychology, Faculty of Psychology and Educational Sciences, Tehran University, Tehran, Iran; 2 Department of Psychology, Islamic Azad University, Bandargaz Branch, Bandargaz, Iran; 3 Faculty of Social Sciences and Education, School of Psychology, Leeds Trinity University, Horsforth, England, UK; 4 Departments of Psychology, Islamic Azad University, Azadshahr Branch, Azadshahr, Iran; 5 Psychology Program, Stockton University, Galloway, NJ USA; 6 Department of Psychology, Faculty of Psychology and Educational Sciences, Abuja University, Abuja, Niger

**Keywords:** Acceptance and commitment therapy, Cancer caregivers, Emotional regulation, Communication avoidance, Hyperarousal, Breast Cancer

## Abstract

**Background:**

Providing care to a family member who has been diagnosed with cancer can be quite tiresome both physically as well as psychologically; therefore, emotional preparedness within the family will contribute significantly in offering effective care. This study investigated the effects of acceptance and commitment therapy (ACT) on communication avoidance, emotional distress and hyper arousal among caregivers of patients with advanced breast cancer.

**Methods:**

This study was a randomized controlled trial with pretest–posttest follow-up design. The study sample were 80 caregivers of advanced breast cancer patients from Mashhad City, Khorasan Province, Iran, in 2023, who were selected by the convenience sampling method. The research instruments included a demographic, communication avoidance, positive and negative affect, and hyperarousal questionnaire. Eight weeks of intervention was conducted following the pre-test. The post-test and follow-up were conducted one week and three month post intervention. The data were analyzed using repeated measures MANOVA.

**Results:**

A statistically significant difference was observed between the intervention and control groups in the mean scores of communication avoidance, positive and negative affect, and hyperarousal questionnaire one week and three month after intervention. The intervention group demonstrated higher scores than the control in positive affect but lower in communication avoidance, negative affect, and hyperarousal. The intervention had a positive effect on the intervention group over time, with no significant differences observed between the post-test and follow-up stages (*p* < .001).

**Conclusion:**

According to these results, ACT was effective in reducing communication avoidance and negative emotions and decreasing hyperarousal among caregivers. Thus, results point to the potential of ACT to contribute not only to emotional regulation but also to healthier coping with the burden associated with providing cancer caregiving.

## Introduction

Globally, cancer is one of the leading causes of death, and many patients have a difficult period when it is diagnosed [1]. This threatening illness brings about a great deal of adverse effects both psychologically and physically for any person during the whole course of cancer [2]. During these hard times, the majority of patients are heavily dependent on the family support system, which includes their care, as well as their emotional and psychological assistance. Due to the nature of cancer and cancer treatments, patients these days often have informal caregivers [3]. Being diagnosed with cancer affects every person who is within that circle, however, not everyone is affected the same way [4–6]. For a large number of people, taking care of an ageing person becomes an unwelcome burden. It has been noted that nearly 50% of family caregivers of cancer patients face either financial problems or sleep deprivation, or psychological consequences such as anxiety or depression [7]. In many Western societies, there is a trend towards individualism, which can be reflected in the caregiving processes. Families often rely on formal healthcare services for support, and caregiving may be shared among various family members without a clear, defined role for each individual [8]. Contrarily, the collective concept and filial piety in many Asian societies make caregiving the sole responsibility of the family, often burdening specific members like the eldest son or daughter-in-law [9]. Improved detection of cancer cases, new treatment modalities, and early integration of palliative care have increased the survival period among cancer patients [10]. Consequently, caregiving, which at one time could have lasted for a matter of weeks or even days has now gone on for several months or even years [11, 12]. The caregivers of patients suffering from advanced cancers perform a barrage of day-to-day activities to ensure that their relatives’ daily living and medical attention are attended to, spending an average of castrating 8 h per day [13]. The tasks encompass aiding with the activities of daily living, organizing care, and controlling the medications and symptoms harmful to the patient [14]. Such life-administering activities are at times massive and may affect the mental health of the different caregivers. This group of people has also been shown to experience increased levels of anxiety, depression, or even illnesses [15, 16]. With respect to the support offered by the caregivers, towards the end of the patient’s life, issues concerning the mental well-being of caregivers become more pronounced. This is due to the fact that they are usually expected to make decisions in relation to the medical care of the patients who are no longer able to do so [17]. Research indicates that approximately one-third family caregivers who take on the responsibility of surrogate decision making suffers from post-traumatic stress syndrome many months to years after the death of the patient [18]. In addition, research shows that the strain brought about by cancer might also interfere with family bonds which in turn affects communication among family members [19, 20].

Past studies have documented the high level of emotional suffering and psychiatric illnesses among cancer patients as well as their loved ones [21]. It has been established previously that 40% of patients, while 30% of spouses, did not talk about recurring cancer with one another [22, 23]. Further, in most instances, family members who provide care fail to pay attention to their own medical requirements, which in turn may affect their well-being [24]. Evidence consistently points to high levels of psychological distress among family caregivers [25, 26]. Furthermore, some studies have shown that patients’ difficulty in adjusting to cancer is significantly correlated with deterioration in family communication [27].

One promising intervention for reducing symptom-related suffering in cancer is Acceptance and Commitment Therapy (ACT) [28, 29]. ACT aims to enhance psychological flexibility, which results in less interference from challenging thoughts, feelings, and symptoms in engaging in meaningful activities [30]. Psychological flexibility is defined as maintaining complete awareness of the present moment while undertaking actions that align with deeply held values [31]. The ACT model identifies six psychological skills—mindfulness, perspective-taking, cognitive diffusion, acceptance, value clarification, and committed action—that promote psychological flexibility [32–34]. ACT enables caregivers to engage more fully in meaningful activities despite the presence of distressing thoughts and feelings [35].

This article explores a new approach to easing the psychological stress experienced by primary caregivers of women with advanced breast cancer. Despite the prevalence of breast cancer in Iran, where it is reported as the most common cancer among women, and the high likelihood of these patients being cared for at home, no significant interventions are planned for their caregivers [26]. This gap in literature is significant, as the existing research predominantly targets pain management and patient-centered interventions, often overlooking the comprehensive needs of the caregivers themselves. The present study aims to address this gap by evaluating the effectiveness of ACT in managing communication avoidance, emotional well-being, and hyperarousal among caregivers. In this regard, further research aims to crystallize the understanding regarding the psychological experiences associated with caregivers, pointing out the problems, as well as therapeutic possibilities. The researchers aim to help caregivers of advanced breast cancer patients to manage communication without compromising their emotional well-being and reducing hyperarousal.

## Materials and methods

### Settings and study design

This study is a randomized controlled trial with a pretest–posttest follow-up design and control group that was conducted over five months between June and October 2023 at a comprehensive cancer center in Mashhad.

*Inclusion criteria*: One of the entry criteria established for the participants was that the primary caregivers had to be over the age of 18 and actively caring for patients with advanced breast cancer. The participants enrolled in the study had to be able to give informed consent and were screened for any type of psychiatric illness or cognitive impairment that could affect their ability to participate in the therapy or in the assessments. In order to maintain the integrity of the study, caregivers who were already taking part in psychological therapy or were undergoing psychiatric medication were excluded from the research.

**Fig. 1:**
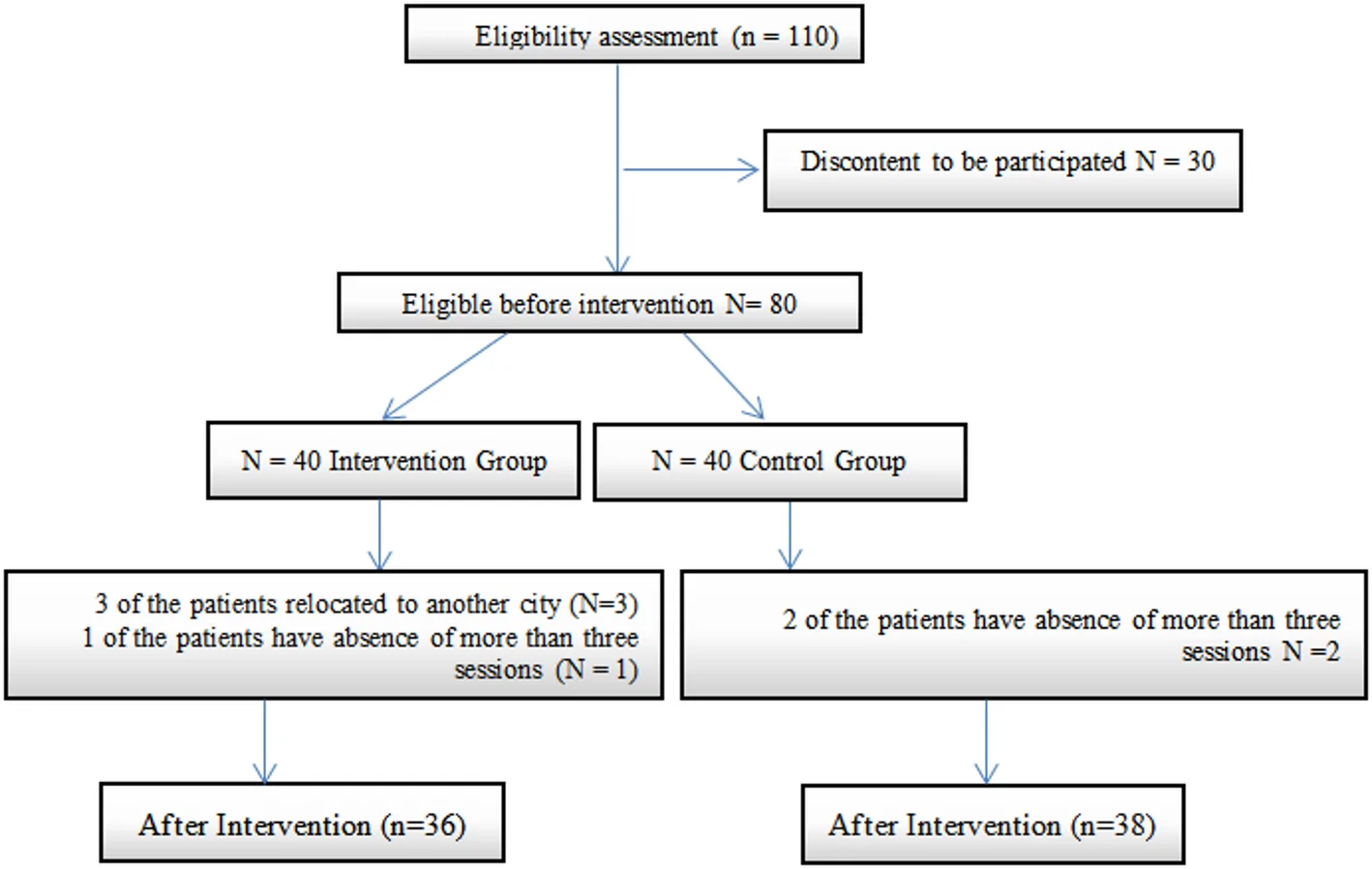
Participants’ flow throughout the study

*Exclusion criteria*: These criteria encompassed caregivers opting to withdraw at any point, those who ceased to meet the study criteria during the trial due to changes in their caregiving status, or those exhibiting severe psychological distress necessitating immediate clinical intervention. From the cancer registry data and clinical staff, 110 breast cancer patients admitted to the cancer center were identified. These patients were contacted to obtain the names of their primary caregivers. In line with this, a total of 80 family caregivers who gave their consent were re-engaged, of whom 68 were spouses/ partners, four were daughters, and eight were sons. The research sample was selected using a convenience sampling method, meaning that only caregivers who were available and willing to participate were included. While this approach facilitated participant recruitment and ensured a sufficient sample size, it inherently introduced selection bias, as the sample may not fully represent the broader population of caregivers. In particular, caregivers who are more available or motivated to participate in psychological interventions may have been overrepresented, potentially impacting the generalizability of the findings. This limitation should be considered when interpreting the results. To mitigate the potential effects of this bias, efforts were made to ensure diversity in the sample in terms of caregivers’ relationships with patients, age, and duration of care. Additionally, random assignment of participants to intervention and control groups helped to balance potential baseline differences. The subjects were then randomly assigned to either an intervention or a control group through simple randomization. Excel’s random number generator was used for randomization and allocation. The study adhered to the Declaration of Helsinki by ensuring respect for participants’ private life and confidentiality. If they agreed to participate and qualified for the study, they were randomly distributed into two groups: experimental and control (*n* = 80). Both groups underwent a pre-test. During the implementation phase, however, a total of six participants withdrew from the study: two from the experimental group due to absence from more than three sessions, and four from the control group (three relocated to another city, and one missed more than three sessions). As a result, the study continued with 74 primary caregivers, comprising 38 in the intervention group and 36 in the control group. See also Fig. 1 on patient participation.

## Measures

### Sociodemographic form

The questionnaires include information on demographic data (age, education, relative, years of living together, work status, etc.).

### Communication avoidance scale

The CAS Questionnaire was constructed for the purpose of estimating the level of communication avoidance in cancer patients and their partners [36]. This evaluation of communication avoidance among cancer patients and their partners consists of 13 items with 10 measuring the verbal form of communication and 3 measuring the non-verbal form. Every single item was measured using a 5 point Likert scale, where 1 represents utmost disagreement and 5 indicates utmost agreement. Previous research has shown the CAS to be reliable (Cronbach alpha = 0.86).

### Positive and negative affect scale (PANAS)

This instrument was developed by Watson. It is a 20-items in length whose first half (10 items) seeks to measure positive affects (like happiness and inspiration) while the rest negative affects (for instance, distress and anxiety) aspired. Each item is rated on a 5-point Likert scale, where 1 is Very slightly or not at All and 5 is Extremely [37]. Its internal consistency was found to be α = 0.79–0.89.

### Hyperarousal scale

The HS was created to measure the post-traumatic stress symptoms experienced within a seven-day period [38]. It comprises of 22 items rated on a Likert scale of 0 (Not at all)—4 (Extremely). This scale will also seek to assess the frequency and severity of the hyperarousal symptoms including: difficulty in maintaining sleep, anger and irritability, attention to an exaggerated level, distractibility, and some somatic complaints. The last week is used for self-exploration of the hyperarousal state in order to understand all the changes that occurred. Previous research has shown the HS to be reliable (Cronbach alpha = 0.80).

### Interventions

The Acceptance and Commitment Therapy program [39] was implemented during the period of two months, August and September 2023. The program had a total of eight sessions taking place in forty minutes each, which were held in a two-week interval. Three months after the initial intervention, a follow-up test was administered to determine the effect several months later, particularly regarding the stability and persistence of the outcomes. This stage proved useful in deciding whether the changes in communication avoidance, negative emotions, hyperarousal, together with an increase in positive emotions in primary caregivers of advanced breast cancer patients due to the Acceptance and Commitment Therapy intervention, were felt long after the intervention. The contents and tasks of the ACT sessions are presented in Table 1.

**Table 1:** Summary of contents of acceptance and commitment therapy program

Sessions	Theme	Home assignments
First Session	Introduction of members, establishing group rules, overview of the therapeutic approach. Tasks included preparing a goal list, prioritizing goals, and assessing methods of controlling disturbing thoughts and emotions.	Participants were encouraged to observe their control methods until the next session.
Second Session	Discussion on the benefits of goal prioritization, examining caregivers’ problems using the ACT framework, focusing on avoidance, fusion, and personal values.	Involved listing pros and cons and control methods for these problems.
Third Session	Review of feedback and the previous session’s homework. Continuation of discussing inclination through ACT metaphors (guest, beggar). Exploration of the ineffectiveness of controlling negative experiences, introduction to values, and the relationship between inclination and values.	Noting significant life areas.
Fourth Session	Review of previous homework, detailed discussion on personal values, identification of internal and external barriers, and introduction to the concept of diffusion (bus metaphor).	Identifying values, goals, actions, and obstacles.
Fifth Session	Review of previous homework, focus on connecting with the present moment (chessboard metaphor), and introduction to mindfulness techniques with a practical exercise.	Recording observations using mindfulness techniques.
Sixth Session	Feedback on mindfulness practices, discussion on self-conceptualization and the use of descriptive versus evaluative language, assessment of life values.	Mindfulness exercises and listing obstacles to achieving values.
Seventh Session	Review of previous tasks, presentation of solutions for overcoming obstacles (vessel of life metaphor), and introduction to reality vs. mind (train of thought metaphor).	Reporting steps towards pursuing values and reflecting on therapy achievements.
Eighth Session	Summarization of therapy concepts, members sharing therapy achievements and future goals. Emphasis on core ACT processes (Acceptance, Diffusion, Self as Context, Being Present, Committed Action), preparation for treatment conclusion, and coping with potential post-treatment challenges.	

### Ethical consideration

To conduct this research, permission was obtained from the Iran National Committee for Ethics in Biomedical research under the code (IR.IAU.ARDABIL.REC.1403.163). After obtaining the necessary permissions from all related departments, sampling was performed. Prior to conducting the study, were provided with detailed explanation about the confidentiality of their information. Subsequently, caregivers completed consent forms.

### Data analysis

All statistical analyses in Study were performed via SPSS 24. Descriptive statistics offered frequencies and percentages in order to present the demographic information of the participants. The effect of Acceptance and Commitment Therapy on Communication Avoidance, Valence, Emotional Expressiveness, and Hyperarousal variables in primary caregivers of women with advanced breast cancer was assayed using multivariate variance analysis. Assumptions of the multivariate variance analysis technique such as normality, equality of variances, homogeneity of regression lines and absence of multicollinearity were considered. The Kolmogorov–Smirnov test was conducted to evaluate the normality of dependent variables such as communication avoidance, positive and negative emotions, and hyperarousal. The z-statistics for these variables were .603, .269, and .108, respectively. As these values were not significant at the .05 level, they suggest a normal distribution of variables within the sample. The M Box test results indicated the homogeneity of the variance-covariance matrix (M BOX, s = 73/34, F = 2.58, *p* = .36 > .05). Furthermore, Levine’s test for equality of variances was non-significant across all variables, supporting the assumption of equal variances between groups. The homogeneity assumption was further supported through the regression homogeneity test as evidenced by the non-significant calculated F-value of the interaction between the group and the pre-test at the .05 level. In addition, the Bonferroni post-hoc test was performed to assess the significant differences of post-test and follow up assessment scores of both experimental and control groups.

## Results

### Participants characteristics

Demographics of the Control Group: With regard to the control group, a majority of the study participants, thirty-two of them (88.89%), were married or had a spouse/partner. In the experimental group, thirty participants (78.95%) were in a spouse/partner relationship. The demographic details are further outlined in Table 2.

**Table 2:** Summary of participant characteristics

Variables		Control (*n* = 36)		Experiment (*n* = 38)	
		N	%	n	%
Relative	Spouse/Partner	32	88.89	30	78.95
	Son/Daughter	4	11.11	8	21.05
Years of living together	1–3 years	6	16.66	9	23.69
	4–10 years	21	58.34	24	63.16
	11 and more	9	25.00	5	13.15
Education level	Primary	3	8.34	4	10.53
	Secondary	22	61.11	27	71.05
	BA	6	16.66	3	7.89
	MA and above	5	13.89	4	10.53
Work status	Employed	32	88.89	34	89.47
	Unemployed	4	11.11	4	10.53
Age	20–30	7	19.44	3	7.89
	31–40	24	66.67	26	68.42
	41 and more	5	13.89	9	23.69
Number of children	1	9	25.00	11	28.94
	2	20	55.55	22	57.90
	3 and more	4	11.11	1	2.63
	No	3	8.34	4	10.53

### Primary outcome

According to Table 2, there is a comparison of the mean scores on avoidance communication, positive emotions, and hyperarousal variables in the experiment group between the pretest and posttest sessions. It was pointed out in particular that the mean value of avoidance communication (21.9 ± 3.1, *p* = .001) as well as of positive emotions (44.7 ± 5.8, *p* = .001) for the experimental group, was significantly higher than the control group. Furthermore, the mean hyperarousal score (9.08 ± 14.92, *p* = .001) measured within the experimental group, was considerably lower than that of the control group. In addition, the results of multivariate analysis of variance with repeated measures of dependent variables (MANOVA) between and within groups revealed significant differences as well. Within the three measuring points of this study—pre-test, post-test, and follow-up—there are significant differences in scores of at least one dependent variable of the study. The Value = .64, F = 101.04, *p* < .001. Also, the groups and time interaction effect-experimental and control groups with time of pre-test, post-test, and follow-up-was significant concerning at least one dependent variable. Value = .32, F = .077, *p* < .001. The specifics are located in the aforementioned Table 3.

**Table 3:** Mean and standard deviation of pre-test, post-test and follow-up scores of variables

Groups		Experimental				Control				Follow-up	
		Pre-test		Post-test		Pre-test		Post-test			
Variables		M	SD	M	SD	M	SD	M	SD	M	SD
Avoidance of communication		38.2	13.2	21.9	3.1	37.1	12.1	34.0	9.09	22.3	4.11
Positive emotions		38.8	5.4	44.7	5.8	37.2	5.5	38.1	6.5	41.64	3.66
Negative emotions		33.7	3.1	24.4	2.9	29.8	3.4	30.9	4.6	23.19	3.46
Hyperarousal		16.3	4.3	9.7	2.3	17.34	5.34	16.1	4.1	9.27	2.73

### Secondary outcome

The results of the repeated measures analysis of variance for the dependent variable scores across the three tests: pre, post, and follow up are displayed in Table 3. The analysis established that the rates on the communication avoidance scale, in particular, reduced significantly from pre-test to post-test (F = 8.64, *p* = .001, *η*^2^ = .35). Moreover, there was no significant difference between the scores of the communication avoidance scale in post-test and follow-up test (F = 5.07, *p* = .01, *η*^2^ = .51). A similar trend was noted in the positive emotion scores, which improved considerably between the pre-test and the post-test (F = 3.56, *p* = .005, *η*^2^ = .36) with no notable differences of those scores in the post-test and at the follow up (F = 11.09, *p* = .13, *η*^2^ = .54). Likewise, the scores for negative emotions also reduced considerably from the pre-test to the post-test (F = 3.56, *p* = .005, *η*^2^ = .36), and the difference in such scores between post-test and follow up was also not significant (F = 11.09, *p* = .13, *η*^2^ = .54). Also, hyperarousal scores decreased from pre-test to post-test level considerably (F = 8.79, *p* < .000, *η*^2^ = .46) but there were no significant changes of such scores between post-test and follow up (F = 18.06, *p* = .38, *η*^2^ = .63). It can be, therefore, deduced that the intervention did not change in its effectiveness during the coaching program course for the subjects within the experimental group. Furthermore, the interaction effect of group and time on the variable of avoidance of communication was found to be significant (F = 46.73, *p* < .000, *η*^2^ = .67) as shown in Table 4. This indicates that avoidance of communication in the experimental group decreased significantly over time. However, no significant difference was observed in the mean scores between the post-test and the follow-up test in the interaction effect of group and time (F = 23.40, *p* = .010, *η*^2^ = .33).

**Table 4:** The results of the analysis of variance with repeated measure of the scores of the dependent variables in the pre-test, post-test, and follow-up stages

Variable	Stage	The sum of the squares	df	Mean of the squares	F	Sig	Effect size
Avoidance of communication	Pre-test–Post-test	423.14	1	423.14	8.64	.001	.35
	Post-test–follow-up	205.11	1	205.11	5.07	.01	.51
Positive emotions	Pre-test–Post-test	33.125	1	31.125	3.56	.005	.36
	Post-test–follow-up	46.182	1	46.182	11.09	.13	.54
Negative emotions	Pre-test–Post-test	379.65	1	379.65	25.08	.000	.47
	Post-test–follow-up	294.02	1	294.02	20.47	.16	.39
Hyperarousal	Pre-test–Post-test	329.19	1	329.19	8.79	.000	.46
	Post-test–follow-up	203.24	1	203.24	18.06	.38	.63

Similarly, the interaction effect of group and time on the variable of positive emotions was significant (F = 11.17, *p* = .00, *η*^2^ = .25). This suggests a significant increase in positive emotions in the experimental group over time. However, in terms of timing, there was no significant difference between the mean scores of the post-test and follow-up test (F = 34.01, *p* = .42, *η*^2^ = .53). The interaction effect with group and time on the aspect of negative emotions was also found to significant (F = 20.1, *p* = .00, *η*^2^ = .91). Therefore, it may be concluded that negative emotions decreased within the experimental group over the length of the study. Nonetheless, in the above-described interactive effect, mean scores obtained in post-test and follow-up test were not found to differ significantly: F = 25.02, *p* = .51, *η*^2^= .78). Similarly, interactions of group and time on the variable hyper arousal were observed to be significant as well: (F = 20.13, *p* = .00 , *η*^2^ = .29) meaning that there was a significant reduction in hyper arousal of the experimental group with time. Yet, the interaction did not yield a statistically significant difference in means in the post-test and follow-up test mean scores: value for F = 32.56, *p* = .19, *η*^2^= .38; which is presented thoroughly in Table 5.

**Table 5:** The results of the analysis of variance with repeated measurements of the scores of dependent variables in the pre-test, post-test, and follow-up stages in the experimental and control groups

Source	Variable	Stage	The sum of the squares	df	The mean of the squares	F	Sig	The effect size
Time * Group	Avoidance of communication	Pre-test–Post-test	1439.09	1	1439.09	46.73	.000	.67
		Post-test–follow-up	946.87	1	946.87	23.40	.010	.33
	Positive emotions	Pre-test–Post-test	614.33	1	614.33	11.17	.00	.25
		Post-test–follow-up	1154.08	1	1154.08	34.01	.42	.53
	Negative emotions	Pre-test–Post-test	148.82	1	148.82	20.1	.00	.91
		Post-test–follow-up	127.70	1	127.70	25.2	.51	.78
	Hyperarousal	Pre-test–Post-test	217.76	1	217.76	20.13	.00	.29
		Post-test–follow-up	103.87	1	103.87	32.56	.19	.38

While analyzing the data using Bonferroni’s post hoc test, it was observed that in the experimental group the mean scores of the dependent variables—communication avoidance, positive emotions, negative emotions, and hyperarousal—from pre-test and post-test periods varied considerably to the advantage of the experimental group in comparison to the control group. On the other hand in the last stage of the study; specifically looking at the mean scores of the dependent variables in the experimental group within the post-test and follow-up periods, distinct from the control group, there were no significant alterations. It was found that there was a noticeable distinction in the between-stage results in the above-mentioned parameters in the courses of pre-test and post-test (as well as pre-test and follow-up) *p* < .001). In contrast, the analysis failed to uncover any difference between post-test and follow up (Table 6).

**Table 6:** The results of bonferroni’s post hoc test

Variables	Groups		Mean differences	*P*
Avoidance of communication	Pre-test	Post-test	− 16.3	0.001
		follow-up	− 15.9	0.001
	Post-test	follow-up	− 0.4	0.135
Positive emotions	Pre-test	Post-test	5.9	0.001
		follow-up	2.26	0.001
	Post-test	follow-up	3.60	0.74
Negative emotions	Pre-test	Post-test	− 9.3	0.001
		follow-up	10.51	0.001
	Post-test	follow-up	1.22	0.139
Hyperarousal	Pre-test	Post-test	− 1.2	0.001
		follow-up	6.03	0.001
	Post-test	follow-up	0.43	0.424

## Discussion

This study presents the evaluation of the efficacy, acceptability, and fidelity of the eight-session ACT‐based group therapy on communication avoidance, emotional well-being, and hyperarousal. Changes over time, between groups, and the interaction between time and group were analyzed using MANOVA analysis. This paper offers results that are, in many respects, encouraging. First, the results showed that ACT not only aided in avoiding communication and reducing hyper arousal but also helped in increasing caregivers’ positive emotions. Second, and most importantly, the effects of the ACT intervention were not only present a short time after treatment was ended (post-test), but they also lasted, as shown by the follow-up tests. This lasting effect points to the favorable long-term outcome of ACT in enhancing the psychological status of the caregivers. This finding is consistent with theoretical predictions in this area as well as the results of previous studies [3, 6, 8, 20, 21].

Moreover, the demographic profile of caregivers in this study, predominantly spouses/partners with a significant proportion in the 31–40 age range and most having secondary education, aligns with previous studies that have similarly identified spouses as the primary caregivers. This demographic trend goes in line with past studies which have established as a fact that middle-aged adults are the main caregivers of people with serious illnesses for instance, those diagnosed with advanced breast cancer [6]. The duration of the relationship, with a notable proportion of caregivers having lived with the patient for 4–11 years, is an interesting aspect that mirrors findings in some earlier studies. These studies have often highlighted the impact of long-term relationships on the caregiving experience, suggesting that longer relationship durations might influence the caregiver’s adaptation and coping mechanisms [23]. The high employment rate among caregivers in both the control and experimental groups is noteworthy and resonates with the increasing trend observed in recent studies, where caregivers often balance employment with their caregiving responsibilities. This dual role can lead to increased stress, a factor that has been extensively studied in caregiver research. These results contribute to the growing body of evidence supporting ACT as a beneficial intervention for caregivers of cancer patients, aligning with previous studies that have reported similar positive outcomes [19]. This finding is crucial as it supports the need for ongoing support and interventions for caregivers beyond the immediate treatment phase, an aspect that has been emphasized in recent research [17].

In ACT, the initial effort is to foster psychological acceptance of mental experiences (thoughts, feelings, etc.) and diminish ineffective control actions. Caregivers realize that attempts to avoid or control their mental experiences often have the opposite effect, intensifying them rather than reducing them. It is recommended to embrace these experiences without any attempts to suppress them internally or externally. Subsequently, the intervention emphasizes psychological presence, fostering awareness of one’s mental states, thoughts, and behaviors in the moment. Caregivers are taught to detach from these mental experiences (cognitive diffusion), enabling them to act independently of them, thereby improving their quality of life and interactions with the patient. ACT trainings assist caregivers in accepting their feelings and symptoms, both physical and psychological. By accepting these feelings, caregivers are less fixated on and reactive to these symptoms. This acceptance facilitates the use of cognitive strategies and the positive regulation of emotions. Furthermore, ACT enhances self-awareness and alertness, both physically and cognitively. This heightened self-awareness promotes accurate self-evaluation and disrupts previously maladaptive thought and feeling patterns, leading caregivers to live more in the moment and experience a positive mental state. The therapy emphasizes accepting the uncontrollable and engaging in activities that make life more fulfilling. The primary aspiration is even when life’s travails are a hindering factor to achieve and maintain a rich, purposeful, and holistic existence. Caregivers learn to embrace their experiences, increase their tolerance, and distance themselves from past experiences and negative thoughts. Employing ACT mechanisms like acceptance, mindfulness, non-judgmental observation of thoughts, and engagement with these thoughts improves emotional regulation, increases psychological flexibility, and enhances the ability to use adaptive strategies.

With regard to the results of this study, several limitations have to be considered. First, caregivers often face time constraints that make attending training classes challenging. This suggests a need for the design of online training classes to accommodate their schedules. Additionally, caregivers require tailored interventions to suit their unique circumstances. A limitation of this study was that all participants received the same standardized intervention. Future research should focus on personalizing treatments to better meet individual needs. The current treatment emphasizes acceptance but does not specifically prepare caregivers for unforeseen situations. So, subsequent interventions should not only aim at conquering individual emotions but also at improving the empowerment skills of the caregivers. Furthermore, given the cultural context of Iran, where emotional expression and family communication patterns may differ from Western contexts, the findings highlight the need to consider cultural adaptations in ACT implementation. While ACT emphasizes mindfulness, presence, and value-based action, its effectiveness in non-Western caregiving settings warrants further exploration.

## Conclusion

This study emphasizes the clinical importance of Acceptance and Commitment Therapy (ACT) in improving the well-being of primary caregivers of women with advanced breast cancer. In addition to its statistical significance, ACT enhances emotional resilience, strengthens coping strategies, and provides a structured approach to sustainable support within healthcare settings. By helping caregivers manage emotional distress, reduce communication avoidance, and alleviate hyperarousal, ACT equips them with practical skills for adaptive caregiving. These findings underscore the necessity of incorporating ACT into psychosocial support programs, particularly in oncology care, where caregivers often experience prolonged stress. Furthermore, ACT positively impacts communication dynamics in cancer-affected families, alleviating anxiety and emotional exhaustion by fostering mindfulness, promoting values-based actions, and enhancing cognitive flexibility, ultimately facilitating open, supportive interactions.

## Data Availability

The data of this study are not publicly available due to ethical/privacy considerations; however, limited information may be provided upon request to the corresponding author.
